# Supplementary material for: Hydroxychloroquine and tocilizumab therapy in COVID-19 patients—An observational study
Source: PLoS One. 2020 Aug 13;15(8):e0237693. doi: 10.1371/journal.pone.0237693 (PMC7425928; doi:10.1371/journal.pone.0237693)
Supplement: S1 Appendix — (DOCX) [file pone.0237693.s001.docx]

Statistical Analysis Plan for Hackensack Meridian Health COVID-19 cases

This plan was pre-specified and is essentially that in the Statistical Analyses section of our manuscript submitted to JAMA. Some details and rationale are included here.

As directed by the editors of JAMA we used propensity-score analyses for all questions addressed (Rosenbaum and Rubin 1984; Austin 2011). We fit a logistic regression model to the probability of being assigned to the experimental arm (hydroxychloroquine, or hydroxychloroquine plus azithromycin, tocilizumab) compared with the control population (cases that were not assigned to the respective treatment). We calculated propensity scores for each patient and stratified patients into propensity-score quintiles. These strata are used as an ordinal covariate to adjust treatment effects in a Cox proportional hazards model.

The model for selecting factors to be included in propensity scores uses a two-stage backward selection approach. We evaluated each of the factors as univariate predictors with factors having p-value less than 0.10 included for further consideration. We removed factors sequentially and one at a time from the multivariate model if their p-values when added to this model were less than 0.50, with largest p-values considered first for removal. We fit the final propensity-scores model using multivariate logistic regression involving all the selected factors as variables in the model. We then stratified propensity scores for the entire population into quintiles and used these quintiles as an ordinal (4-degree-of-freedom) variable.

The only endpoint we considered was mortality. We used the ordinal quintile propensity-score variable as a covariate in a Cox proportional-hazards model to adjust the relative treatment comparison in a proportional hazards model was fit using the corresponding quintile stratification for adjustment. (The output of these models using JMP® Pro 15.0.0. is shown in supplementary material.)

We considered the following factors for all propensity-score models: gender, coronary disease, stroke, heart failure, arrhythmia, African American, COPD, coronary disease, stroke, heart failure, arrhythmia, renal failure, rheumatologic disorder, inflammatory bowel disease, advanced liver disease, age, diabetes, insulin, asthma, HIV/hepatitis, cancer, and log ferritin. The final propensity-score model for hydroxychloroquine included the first 15 of these factors. That for hydroxychloroquine plus azithromycin included the first 5 of these factors plus cancer. That for tocilizumab included age, gender, COPD, and renal failure.

We knew that hydroxychloroquine or hydroxychloroquine plus azithromycin had been assigned to the majority of patients, with initial dose usually early in their hospital stay. For these two regimens we used the start date as the day of hospital admission, whether they eventually were admitted to the ICU or not. The control population consisted of patients who never received the respective regimen. So there was overlap in these two control populations.

We used an additional analysis to facilitate comparison with Figure 2 of Rosenberg et al. (2020). Namely, we added an analysis of the factorial nature of treatment with hydroxychloroquine and azithromycin. Figure 2 from this article showed Kaplan-Meier curves for hydroxychloroquine + azithromycin, hydroxychloroquine alone, azithromycin alone, and neither drug. However, there is no natural way to generalize propensity scores that are designed to predict particular therapies and to a factorial setting. Therefore, for these analyses we pre-specified that we would use the average of the two propensity scores calculated separately for hydroxychloroquine and hydroxychloroquine plus azithromycin. Then we stratified into propensity quintiles based on that average and proceeded as indicated above.

We knew that the use of tocilizumab in the hospital was very different than that for hydroxychloroquine and azithromycin. Firstly, it had been assigned to only a small minority of patients. In addition, it had been assigned preferentially to patients in the ICU. Of the patients who received tocilizumab at any time and were admitted to the ICU at some time during their hospital stay, only 11 had received their first dose of tocilizumab before entering the ICU. We excluded these patients from the tocilizumab analysis. The control patients for the tocilizumab comparison were those who had been admitted to the ICU at some time during their hospital stay but who had not received tocilizumab either before or after admission to the ICU. The start time for the tocilizumab analysis was the day of admission to the ICU. The propensity-score analysis described above for hydroxychloroquine was used for tocilizumab as well, but only four factors met the predefined conditions for inclusion in the propensity score.

Patients still alive and in the hospital were censored as of May 5, 2020. Patients who had been discharged from the hospital were censored as of day 36 following hospital admission.

Based on the Cox proportional regression model using the propensity-score quintiles and treatment, we provide hazard ratios (treatment in comparison with control) together with corresponding p-values and confidence intervals based on Wald tests, and include these analyses in Supplementary Material. We also show the survival data using Kaplan-Meier plots unadjusted for propensity scores. From these Kaplan-Meier curves we identify 30-day mortality for each treatment and control considered.

Confidence intervals and p-values in this study are descriptive measures of distance between outcomes of treatment groups or distance from hazard ratio 1.00. These measures do not have the same inferential interpretations that are possible for primary endpoint analyses of RCTs.

Rosenbaum, PR, Rubin DB. Reducing bias in observational studies using subclassification on the propensity score. *Journal of the American Statistical Association*, 79(1984):516-524.

Austin PC. An introduction to propensity score methods for reducing the effects of confounding in observational studies. *Multivariate Behavioral Research*, 46(2011):399– 424.

Rosenberg ES, Dufort EM, Udo T, et al. Association of treatment with hydroxychloroquine or azithromycin with in-hospital mortality in patients with COVID-19 in NewYork State. *JAMA*. doi:10.1001/jama.2020.8630. Published online May 11, 2020.
