# Supplementary material for: Hydroxychloroquine and tocilizumab therapy in COVID-19 patients—An observational study
Source: PLoS One. 2020 Aug 13;15(8):e0237693. doi: 10.1371/journal.pone.0237693 (PMC7425928; doi:10.1371/journal.pone.0237693)
Supplement: S2 Appendix — (DOCX) [file pone.0237693.s002.docx]

# Statistical Output

JMP® Pro 15.0.0 output for proportional hazards analyses adjusting for propensity-score quintiles (called buckets) plus associate Kaplan-Meier plots

Pages 1 to 7: Tocilizumab

Pages 8 to 14: Hydroxychloroquine (HCQ) Pages 15 to 21: HCQ + azithromycin (AZI) Pages 22 to 30: HCQ & AZI factorial

Tocilizumab Proportional Hazards Fit

Censored By: Censor


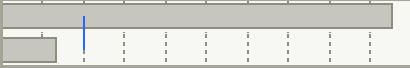
Effect Summary

| **Source** | **LogWorth** | **PValue** |
| --- | --- | --- |
| Bucket | 9.544 | 0.00000 |
| toci | 1.326 | 0.04721 |

| **Whole Model** |  |
| --- | --- |
| Number of Events | 297 |
| Number of Censorings | 250 |
| Total Number | 547 |

AICc BIC

3497.7 3519.11

| **Model** | **-LogLikelihood** | **ChiSquare** | **DF** | **Prob>Chisq** |
| --- | --- | --- | --- | --- |
| Difference | 28.739 | 57.4784 | 5 | <.0001* |
| Full | 1743.794 |  |  |  |
| Reduced | 1772.534 |  |  |  |

Parameter Estimates

| **Term** | **Estimate** | **Std Error** | **Lower 95%** | **Upper 95%** |
| --- | --- | --- | --- | --- |
| Bucket[1] | 0.60403196 | 0.1060684 | 0.392189 | 0.8085971 |
| Bucket[2] | 0.22226637 | 0.1113988 | -0.001307 | 0.436129 |
| Bucket[3] | 0.02744193 | 0.1163965 | -0.207025 | 0.2501206 |
| Bucket[4] | -0.11639 | 0.1226699 | -0.364581 | 0.1173186 |
| toci[FALSE] | 0.1395509 | 0.072186 | 0.0016758 | 0.285154 |

Confidence Intervals are profile-likelihood

Baseline Survival at mean


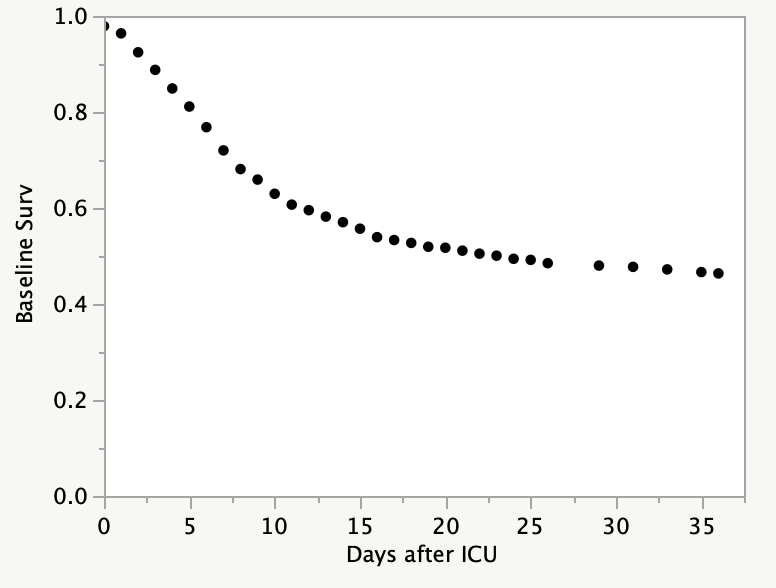


Effect Likelihood Ratio Tests

| **Source** | **Nparm** | **DF** | **L-R**  **ChiSquare** | **Prob>ChiSq** |
| --- | --- | --- | --- | --- |
| Bucket | 4 | 4 | 50.4873372 | <.0001* |
| toci | 1 | 1 | 3.93779952 | 0.0472* |

Effect Wald Tests

| **Source** | **Nparm** | **DF** | **Wald ChiSquare** | **Prob>ChiSq** |
| --- | --- | --- | --- | --- |
| Bucket | 4 | 4 | 46.7013729 | <.0001* |
| toci | 1 | 1 | 3.73731167 | 0.0532 |

Risk Ratios

Risk Ratios for Bucket

| **Level1** | **/Level2** | **Risk Ratio** | **Prob>Chisq** | **Lower 95%** | **Upper 95%** |
| --- | --- | --- | --- | --- | --- |
| 2 | 1 | 0.6826551 | 0.0200* | 0.4949178 | 0.9416067 |
| 3 | 1 | 0.5618109 | 0.0007* | 0.402412 | 0.784349 |
| 3 | 2 | 0.8229791 | 0.2681 | 0.5829601 | 1.1618198 |
| 4 | 1 | 0.4865469 | <.0001* | 0.3438065 | 0.6885496 |
| 4 | 2 | 0.7127273 | 0.0638 | 0.4981762 | 1.0196797 |
| 4 | 3 | 0.8660332 | 0.4441 | 0.5991581 | 1.2517791 |
| 5 | 1 | 0.261484 | <.0001* | 0.1739388 | 0.3930916 |
| 5 | 2 | 0.3830397 | <.0001* | 0.2526309 | 0.580766 |
| 5 | 3 | 0.4654307 | 0.0004* | 0.3046074 | 0.7111635 |
| 5 | 4 | 0.5374282 | 0.0052* | 0.3475804 | 0.8309705 |
| 1 | 2 | 1.4648687 | 0.0200* | 1.0620146 | 2.0205375 |
| 1 | 3 | 1.7799585 | 0.0007* | 1.2749427 | 2.4850154 |
| 2 | 3 | 1.2150976 | 0.2681 | 0.8607187 | 1.7153831 |
| 1 | 4 | 2.0553004 | <.0001* | 1.4523281 | 2.9086125 |
| 2 | 4 | 1.4030612 | 0.0638 | 0.9807001 | 2.0073219 |
| 3 | 4 | 1.1546901 | 0.4441 | 0.798863 | 1.6690086 |
| 1 | 5 | 3.8243258 | <.0001* | 2.5439361 | 5.7491489 |
| 2 | 5 | 2.6106953 | <.0001* | 1.7218638 | 3.9583444 |
| 3 | 5 | 2.1485478 | 0.0004* | 1.4061464 | 3.2829139 |
| 4 | 5 | 1.8607138 | 0.0052* | 1.2034122 | 2.8770323 |

Risk Ratios for toci

| **Level1** | **/Level2** | **Risk Ratio** | **Prob>Chisq** | **Lower 95%** | **Upper 95%** |
| --- | --- | --- | --- | --- | --- |
| TRUE | FALSE | 0.7564629 | 0.0532 | 0.5700303 | 1.0038696 |
| FALSE | TRUE | 1.3219419 | 0.0532 | 0.9961453 | 1.7542926 |

Normal approximations used for ratio confidence limits effects: Bucket toci

Product-Limit Survival Fit Survival Plot


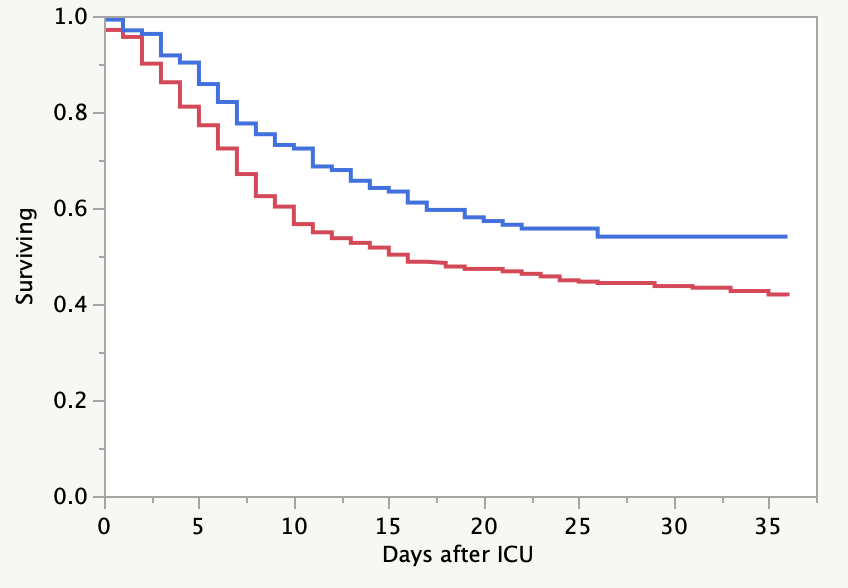

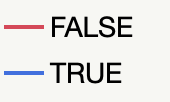


Time to event: Days after ICU Censored by Censor

Censor Code 1 Grouped by toci

| **Summary** |  | | | |
| --- | --- | --- | --- | --- |
| **Group** | **Number failed** | **Number censored** | **Mean** | **Std Error** |
| FALSE | 236 | 177 | 20.1264 | 0.724 |
| TRUE | 61 | 73 | 18.4596 Biased | 0.8058 |
| Combined | 297 | 250 | 21.0465 | 0.62448 |

| **Quantiles** |  | | |
| --- | --- | --- | --- |
| **Group Median Time** | **Lower 95% Upper 95%** | **25%**  **Failures** | **75%**  **Failures** |
| FALSE 16 | 12 24 | 6 | . |
| TRUE . | 19 . | 9 | . |
| Combined 19 | 15 33 | 6 | . |
| **Tests Between Groups** |  |  |  |
| **Test ChiSquare** | **DF Prob>ChiSq** |  |  |
| Log-Rank 6.8569 | 1 0.0088* |  |  |
| Wilcoxon 7.6214 | 1 0.0058* |  |  |

FALSE (non toci)

| **Days after ICU** | **Survival** | **Failure** | **SurvStdErr** | **Number failed** | **Number censored** | **At Risk** |
| --- | --- | --- | --- | --- | --- | --- |
| 0.0000 | 1.0000 | 0.0000 | 0.0000 | 0 | 0 | 413 |
| 0.0000 | 0.9709 | 0.0291 | 0.0083 | 12 | 0 | 413 |
| 1.0000 | 0.9564 | 0.0436 | 0.0100 | 6 | 0 | 401 |
| 2.0000 | 0.9007 | 0.0993 | 0.0147 | 23 | 0 | 395 |
| 3.0000 | 0.8620 | 0.1380 | 0.0170 | 16 | 0 | 372 |
| 4.0000 | 0.8111 | 0.1889 | 0.0193 | 21 | 0 | 356 |
| 5.0000 | 0.7724 | 0.2276 | 0.0206 | 16 | 0 | 335 |
| 6.0000 | 0.7240 | 0.2760 | 0.0220 | 20 | 0 | 319 |
| 7.0000 | 0.6707 | 0.3293 | 0.0231 | 22 | 0 | 299 |
| 8.0000 | 0.6247 | 0.3753 | 0.0238 | 19 | 0 | 277 |
| 9.0000 | 0.6029 | 0.3971 | 0.0241 | 9 | 1 | 258 |
| 10.0000 | 0.5664 | 0.4336 | 0.0244 | 15 | 1 | 248 |
| 11.0000 | 0.5493 | 0.4507 | 0.0245 | 7 | 0 | 232 |
| 12.0000 | 0.5371 | 0.4629 | 0.0246 | 5 | 0 | 225 |
| 13.0000 | 0.5274 | 0.4726 | 0.0246 | 4 | 1 | 220 |
| 14.0000 | 0.5176 | 0.4824 | 0.0246 | 4 | 1 | 215 |
| 15.0000 | 0.5028 | 0.4972 | 0.0246 | 6 | 0 | 210 |
| 16.0000 | 0.4880 | 0.5120 | 0.0246 | 6 | 0 | 204 |
| 17.0000 | 0.4855 | 0.5145 | 0.0246 | 1 | 1 | 198 |
| 18.0000 | 0.4781 | 0.5219 | 0.0246 | 3 | 1 | 196 |
| 19.0000 | 0.4731 | 0.5269 | 0.0246 | 2 | 2 | 192 |
| 20.0000 | 0.4731 | 0.5269 | 0.0246 | 0 | 1 | 188 |

| **Days after ICU** | **Survival** | **Failure** | **SurvStdErr** | **Number failed** | **Number censored** | **At Risk** |
| --- | --- | --- | --- | --- | --- | --- |
| 21.0000 | 0.4681 | 0.5319 | 0.0246 | 2 | 5 | 187 |
| 22.0000 | 0.4629 | 0.5371 | 0.0246 | 2 | 5 | 180 |
| 23.0000 | 0.4575 | 0.5425 | 0.0246 | 2 | 2 | 173 |
| 24.0000 | 0.4494 | 0.5506 | 0.0246 | 3 | 6 | 169 |
| 25.0000 | 0.4466 | 0.5534 | 0.0246 | 1 | 2 | 160 |
| 26.0000 | 0.4437 | 0.5563 | 0.0246 | 1 | 6 | 157 |
| 27.0000 | 0.4437 | 0.5563 | 0.0246 | 0 | 5 | 150 |
| 28.0000 | 0.4437 | 0.5563 | 0.0246 | 0 | 6 | 145 |
| 29.0000 | 0.4373 | 0.5627 | 0.0247 | 2 | 4 | 139 |
| 30.0000 | 0.4373 | 0.5627 | 0.0247 | 0 | 4 | 133 |
| 31.0000 | 0.4340 | 0.5660 | 0.0247 | 1 | 2 | 129 |
| 33.0000 | 0.4271 | 0.5729 | 0.0248 | 2 | 5 | 126 |
| 34.0000 | 0.4271 | 0.5729 | 0.0248 | 0 | 1 | 119 |
| 35.0000 | 0.4198 | 0.5802 | 0.0249 | 2 | 1 | 118 |
| 36.0000 | 0.4162 | 0.5838 | 0.0250 | 1 | 114 | 115 |

TRUE (toci)

| **Days after ICU** | **Survival** | **Failure** | **SurvStdErr** | **Number failed** | **Number censored** | **At Risk** |
| --- | --- | --- | --- | --- | --- | --- |
| 0.0000 | 1.0000 | 0.0000 | 0.0000 | 0 | 0 | 134 |
| 0.0000 | 0.9925 | 0.0075 | 0.0074 | 1 | 0 | 134 |
| 1.0000 | 0.9701 | 0.0299 | 0.0147 | 3 | 0 | 133 |
| 2.0000 | 0.9627 | 0.0373 | 0.0164 | 1 | 0 | 130 |
| 3.0000 | 0.9179 | 0.0821 | 0.0237 | 6 | 0 | 129 |
| 4.0000 | 0.9030 | 0.0970 | 0.0256 | 2 | 0 | 123 |
| 5.0000 | 0.8582 | 0.1418 | 0.0301 | 6 | 0 | 121 |
| 6.0000 | 0.8209 | 0.1791 | 0.0331 | 5 | 0 | 115 |
| 7.0000 | 0.7761 | 0.2239 | 0.0360 | 6 | 0 | 110 |
| 8.0000 | 0.7537 | 0.2463 | 0.0372 | 3 | 0 | 104 |
| 9.0000 | 0.7313 | 0.2687 | 0.0383 | 3 | 0 | 101 |
| 10.0000 | 0.7239 | 0.2761 | 0.0386 | 1 | 0 | 98 |
| 11.0000 | 0.6866 | 0.3134 | 0.0401 | 5 | 0 | 97 |
| 12.0000 | 0.6791 | 0.3209 | 0.0403 | 1 | 0 | 92 |
| 13.0000 | 0.6567 | 0.3433 | 0.0410 | 3 | 0 | 91 |
| 14.0000 | 0.6418 | 0.3582 | 0.0414 | 2 | 1 | 88 |
| 15.0000 | 0.6342 | 0.3658 | 0.0416 | 1 | 1 | 85 |
| 16.0000 | 0.6113 | 0.3887 | 0.0422 | 3 | 0 | 83 |

| **Days after ICU** | **Survival** | **Failure** | **SurvStdErr** | **Number failed** | **Number censored** | **At Risk** |
| --- | --- | --- | --- | --- | --- | --- |
| 17.0000 | 0.5960 | 0.4040 | 0.0425 | 2 | 1 | 80 |
| 19.0000 | 0.5806 | 0.4194 | 0.0428 | 2 | 0 | 77 |
| 20.0000 | 0.5728 | 0.4272 | 0.0429 | 1 | 0 | 75 |
| 21.0000 | 0.5651 | 0.4349 | 0.0430 | 1 | 1 | 74 |
| 22.0000 | 0.5572 | 0.4428 | 0.0431 | 1 | 0 | 72 |
| 23.0000 | 0.5572 | 0.4428 | 0.0431 | 0 | 1 | 71 |
| 24.0000 | 0.5572 | 0.4428 | 0.0431 | 0 | 2 | 70 |
| 25.0000 | 0.5572 | 0.4428 | 0.0431 | 0 | 2 | 68 |
| 26.0000 | 0.5403 | 0.4597 | 0.0434 | 2 | 0 | 66 |
| 30.0000 | 0.5403 | 0.4597 | 0.0434 | 0 | 2 | 64 |
| 34.0000 | 0.5403 | 0.4597 | 0.0434 | 0 | 1 | 62 |
| 36.0000 | 0.5403 | 0.4597 | 0.0434 | 0 | 61 | 61 |

HCQ Proportional Hazards Fit

Censored By: Censor


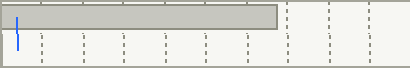
Effect Summary

| **Source** | **LogWorth** | | **PValue** |
| --- | --- | --- | --- |
| HCQ Buckets | 33.890 | | 0.00000 |
| Any HCQ | 0.038 | | 0.91653 |
| **Whole Model** |  | |  |
| Number of Events | | 491 | |
| Number of Censorings | | 2021 | |
| Total Number | | 2512 | |

AICc BIC

7406.48 7435.6

| **Model** | **-LogLikelihood** | **ChiSquare** | **DF** | **Prob>Chisq** |
| --- | --- | --- | --- | --- |
| Difference | 82.724 | 165.4488 | 5 | <.0001* |
| Full | 3698.230 |  |  |  |
| Reduced | 3780.954 |  |  |  |

Parameter Estimates

| **Term** | **Estimate** | **Std Error** | **Lower 95%** | **Upper 95%** |
| --- | --- | --- | --- | --- |
| HCQ Buckets[2-1] | -0.8281712 | 0.1339532 | -1.090715 | -0.565628 |
| HCQ Buckets[3-2] | -0.1105759 | 0.1587663 | -0.421752 | 0.2006002 |
| HCQ Buckets[4-3] | -1.0549883 | 0.2212807 | -1.488691 | -0.621286 |
| HCQ Buckets[5-4] | 1.68381111 | 0.207857 | 1.2764188 | 2.0912034 |
| Any HCQ[No] | 0.00551589 | 0.0525815 | -0.097542 | 0.1085737 |

Confidence Intervals are Wald

Baseline Survival at mean


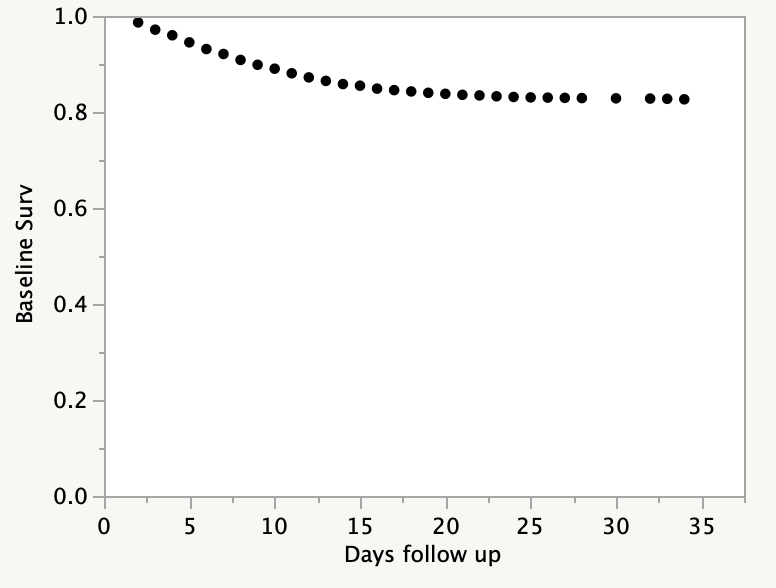


Effect Likelihood Ratio Tests

| **Source** | **Nparm** | **DF** | **L-R**  **ChiSquare** | **Prob>ChiSq** |
| --- | --- | --- | --- | --- |
| HCQ Buckets | 4 | 4 | 164.916681 | <.0001* |
| Any HCQ | 1 | 1 | 0.01098421 | 0.9165 |

Effect Wald Tests

| **Source** | **Nparm** | **DF** | **Wald ChiSquare** | **Prob>ChiSq** |
| --- | --- | --- | --- | --- |
| HCQ Buckets | 4 | 4 | 132.93464 | <.0001* |
| Any HCQ | 1 | 1 | 0.01100438 | 0.9165 |

Risk Ratios

Risk Ratios for HCQ Buckets

| **Level1** | **/Level2** | **Risk Ratio** | **Prob>Chisq** | **Lower 95%** | **Upper 95%** |
| --- | --- | --- | --- | --- | --- |
| 2 | 1 | 0.4368474 | <.0001* | 0.3359763 | 0.5680035 |
| 3 | 1 | 0.3911175 | <.0001* | 0.2984022 | 0.5126401 |
| 3 | 2 | 0.8953183 | 0.4861 | 0.6558966 | 1.2221361 |
| 4 | 1 | 0.1361858 | <.0001* | 0.0912441 | 0.2032632 |
| 4 | 2 | 0.3117467 | <.0001* | 0.2030396 | 0.4786555 |
| 4 | 3 | 0.3481965 | <.0001* | 0.225668 | 0.537253 |
| 5 | 1 | 0.7335024 | 0.0075* | 0.5844893 | 0.9205058 |
| 5 | 2 | 1.6790814 | 0.0002* | 1.276406 | 2.2087912 |
| 5 | 3 | 1.8754016 | <.0001* | 1.4147885 | 2.4859765 |
| 5 | 4 | 5.3860437 | <.0001* | 3.5837825 | 8.0946505 |
| 1 | 2 | 2.2891286 | <.0001* | 1.7605526 | 2.9764007 |
| 1 | 3 | 2.5567762 | <.0001* | 1.9506863 | 3.351182 |
| 2 | 3 | 1.1169212 | 0.4861 | 0.8182395 | 1.5246305 |
| 1 | 4 | 7.3429119 | <.0001* | 4.9197294 | 10.959618 |
| 2 | 4 | 3.2077323 | <.0001* | 2.0891853 | 4.925148 |
| 3 | 4 | 2.8719416 | <.0001* | 1.8613204 | 4.4312889 |
| 1 | 5 | 1.363322 | 0.0075* | 1.0863593 | 1.7108952 |
| 2 | 5 | 0.5955637 | 0.0002* | 0.4527363 | 0.7834498 |
| 3 | 5 | 0.5332191 | <.0001* | 0.4022564 | 0.7068194 |
| 4 | 5 | 0.185665 | <.0001* | 0.1235384 | 0.2790348 |

Risk Ratios for Any HCQ

| **Level1** | **/Level2** | **Risk Ratio** | **Prob>Chisq** | **Lower 95%** | **Upper 95%** |
| --- | --- | --- | --- | --- | --- |
| Yes | No | 0.9890288 | 0.9165 | 0.8048113 | 1.215413 |
| No | Yes | 1.0110929 | 0.9165 | 0.8227656 | 1.2425273 |

Normal approximations used for ratio confidence limits effects: HCQ Buckets Any HCQ

Product-Limit Survival Fit Survival Plot


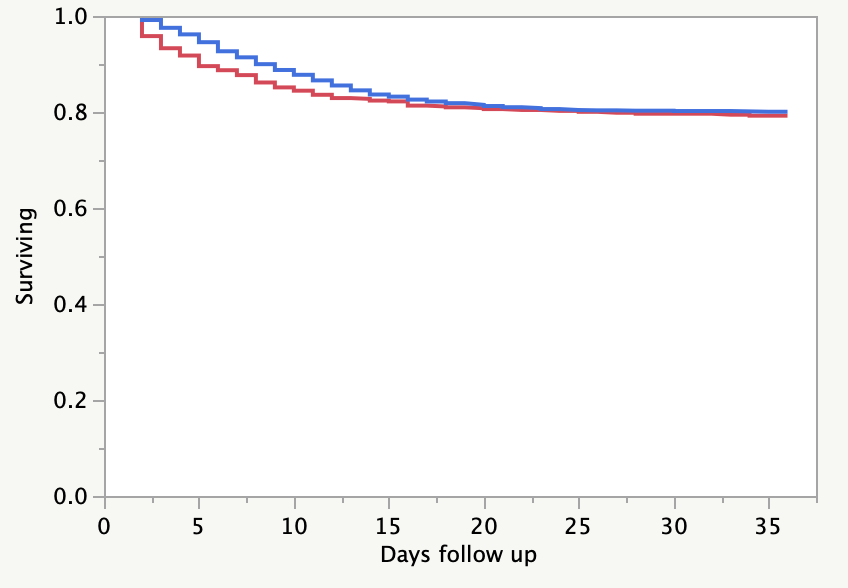

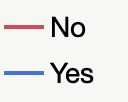


Time to event: Days follow up Censored by Censor

Censor Code 1 Grouped by Any HCQ

Summary

| **Group** | **Number failed** | **Number censored** | **Mean** | **Std Error** |
| --- | --- | --- | --- | --- |
| No | 121 | 477 | 28.5965 Biased | 0.45535 |
| Yes | 370 | 1544 | 29.1582 Biased | 0.23204 |
| Combined | 491 | 2021 | 29.0243 Biased | 0.20721 |

| **Quantiles** |  | | | | |
| --- | --- | --- | --- | --- | --- |
| **Group** | **Median Time** | **Lower 95%** | **Upper 95%** | **25%**  **Failures** | **75%**  **Failures** |
| No | . | . | . | . | . |
| Yes | . | . | . | . | . |
| Combined | . | . | . | . | . |

Tests Between Groups

| **Test** | **ChiSquare** | **DF** | **Prob>ChiSq** |  | |
| --- | --- | --- | --- | --- | --- |
| Log-Rank | 0.5463 | 1 | 0.4598 |  |  |
| Wilcoxon | 1.1488 | 1 | 0.2838 |  |  |
| **No** |  |  |  |  |  |
| **Days follow up** | **Survival** | **Failure** | **SurvStdErr** | **Number Number failed censored** | **At Risk** |
| 0.0000 | 1.0000 | 0.0000 | 0.0000 | 0 0 | 598 |
| 1.0000 | 1.0000 | 0.0000 | 0.0000 | 0 1 | 598 |
| 2.0000 | 0.9581 | 0.0419 | 0.0082 | 25 3 | 597 |
| 3.0000 | 0.9329 | 0.0671 | 0.0103 | 15 3 | 569 |
| 4.0000 | 0.9176 | 0.0824 | 0.0113 | 9 1 | 551 |
| 5.0000 | 0.8956 | 0.1044 | 0.0126 | 13 2 | 541 |
| 6.0000 | 0.8871 | 0.1129 | 0.0130 | 5 0 | 526 |
| 7.0000 | 0.8768 | 0.1232 | 0.0135 | 6 1 | 521 |
| 8.0000 | 0.8615 | 0.1385 | 0.0142 | 9 1 | 514 |
| 9.0000 | 0.8512 | 0.1488 | 0.0146 | 6 0 | 504 |
| 10.0000 | 0.8444 | 0.1556 | 0.0149 | 4 0 | 498 |
| 11.0000 | 0.8359 | 0.1641 | 0.0152 | 5 1 | 494 |
| 12.0000 | 0.8290 | 0.1710 | 0.0155 | 4 2 | 488 |
| 13.0000 | 0.8273 | 0.1727 | 0.0156 | 1 4 | 482 |
| 14.0000 | 0.8238 | 0.1762 | 0.0157 | 2 3 | 477 |
| 15.0000 | 0.8221 | 0.1779 | 0.0158 | 1 3 | 472 |
| 16.0000 | 0.8133 | 0.1867 | 0.0161 | 5 6 | 468 |
| 17.0000 | 0.8115 | 0.1885 | 0.0161 | 1 1 | 457 |
| 18.0000 | 0.8097 | 0.1903 | 0.0162 | 1 6 | 455 |
| 19.0000 | 0.8079 | 0.1921 | 0.0163 | 1 7 | 448 |
| 20.0000 | 0.8061 | 0.1939 | 0.0163 | 1 5 | 440 |
| 21.0000 | 0.8042 | 0.1958 | 0.0164 | 1 7 | 434 |

| **Days follow up** | **Survival** | **Failure** | **SurvStdErr** | **Number failed** | **Number censored** | **At Risk** |
| --- | --- | --- | --- | --- | --- | --- |
| 22.0000 | 0.8042 | 0.1958 | 0.0164 | 0 | 6 | 426 |
| 23.0000 | 0.8023 | 0.1977 | 0.0165 | 1 | 4 | 420 |
| 24.0000 | 0.8023 | 0.1977 | 0.0165 | 0 | 2 | 415 |
| 25.0000 | 0.8004 | 0.1996 | 0.0165 | 1 | 3 | 413 |
| 26.0000 | 0.7984 | 0.2016 | 0.0166 | 1 | 7 | 409 |
| 27.0000 | 0.7984 | 0.2016 | 0.0166 | 0 | 1 | 401 |
| 28.0000 | 0.7964 | 0.2036 | 0.0167 | 1 | 4 | 400 |
| 29.0000 | 0.7964 | 0.2036 | 0.0167 | 0 | 1 | 395 |
| 30.0000 | 0.7964 | 0.2036 | 0.0167 | 0 | 2 | 394 |
| 31.0000 | 0.7964 | 0.2036 | 0.0167 | 0 | 2 | 392 |
| 32.0000 | 0.7944 | 0.2056 | 0.0168 | 1 | 3 | 390 |
| 33.0000 | 0.7944 | 0.2056 | 0.0168 | 0 | 1 | 386 |
| 34.0000 | 0.7923 | 0.2077 | 0.0169 | 1 | 2 | 385 |
| 35.0000 | 0.7923 | 0.2077 | 0.0169 | 0 | 2 | 382 |
| 36.0000 | 0.7923 | 0.2077 | 0.0169 | 0 | 380 | 380 |

Yes

| **Days follow up** | **Survival** | **Failure** | **SurvStdErr** | **Number failed** | **Number censored** | **At Risk** |
| --- | --- | --- | --- | --- | --- | --- |
| 0.0000 | 1.0000 | 0.0000 | 0.0000 | 0 | 0 | 1914 |
| 0.0000 | 1.0000 | 0.0000 | 0.0000 | 0 | 1 | 1914 |
| 1.0000 | 1.0000 | 0.0000 | 0.0000 | 0 | 2 | 1913 |
| 2.0000 | 0.9916 | 0.0084 | 0.0021 | 16 | 3 | 1911 |
| 3.0000 | 0.9754 | 0.0246 | 0.0035 | 31 | 3 | 1892 |
| 4.0000 | 0.9617 | 0.0383 | 0.0044 | 26 | 2 | 1858 |
| 5.0000 | 0.9454 | 0.0546 | 0.0052 | 31 | 0 | 1830 |
| 6.0000 | 0.9265 | 0.0735 | 0.0060 | 36 | 2 | 1799 |
| 7.0000 | 0.9139 | 0.0861 | 0.0064 | 24 | 3 | 1761 |
| 8.0000 | 0.8997 | 0.1003 | 0.0069 | 27 | 1 | 1734 |
| 9.0000 | 0.8875 | 0.1125 | 0.0072 | 23 | 3 | 1706 |
| 10.0000 | 0.8775 | 0.1225 | 0.0075 | 19 | 2 | 1680 |
| 11.0000 | 0.8659 | 0.1341 | 0.0078 | 22 | 7 | 1659 |
| 12.0000 | 0.8552 | 0.1448 | 0.0081 | 20 | 8 | 1630 |
| 13.0000 | 0.8451 | 0.1549 | 0.0083 | 19 | 19 | 1602 |
| 14.0000 | 0.8364 | 0.1636 | 0.0085 | 16 | 32 | 1564 |
| 15.0000 | 0.8320 | 0.1680 | 0.0086 | 8 | 28 | 1516 |
| 16.0000 | 0.8258 | 0.1742 | 0.0087 | 11 | 16 | 1480 |

| **Days follow up** | **Survival** | **Failure** | **SurvStdErr** | **Number failed** | **Number censored** | **At Risk** |
| --- | --- | --- | --- | --- | --- | --- |
| 17.0000 | 0.8219 | 0.1781 | 0.0088 | 7 | 18 | 1453 |
| 18.0000 | 0.8184 | 0.1816 | 0.0089 | 6 | 33 | 1428 |
| 19.0000 | 0.8149 | 0.1851 | 0.0090 | 6 | 36 | 1389 |
| 20.0000 | 0.8125 | 0.1875 | 0.0090 | 4 | 29 | 1347 |
| 21.0000 | 0.8100 | 0.1900 | 0.0091 | 4 | 35 | 1314 |
| 22.0000 | 0.8081 | 0.1919 | 0.0091 | 3 | 34 | 1275 |
| 23.0000 | 0.8061 | 0.1939 | 0.0092 | 3 | 26 | 1238 |
| 24.0000 | 0.8041 | 0.1959 | 0.0092 | 3 | 20 | 1209 |
| 25.0000 | 0.8034 | 0.1966 | 0.0092 | 1 | 15 | 1186 |
| 26.0000 | 0.8034 | 0.1966 | 0.0092 | 0 | 23 | 1170 |
| 27.0000 | 0.8027 | 0.1973 | 0.0093 | 1 | 10 | 1147 |
| 28.0000 | 0.8027 | 0.1973 | 0.0093 | 0 | 15 | 1136 |
| 29.0000 | 0.8027 | 0.1973 | 0.0093 | 0 | 7 | 1121 |
| 30.0000 | 0.8020 | 0.1980 | 0.0093 | 1 | 9 | 1114 |
| 31.0000 | 0.8020 | 0.1980 | 0.0093 | 0 | 3 | 1104 |
| 32.0000 | 0.8020 | 0.1980 | 0.0093 | 0 | 9 | 1101 |
| 33.0000 | 0.8013 | 0.1987 | 0.0093 | 1 | 8 | 1092 |
| 34.0000 | 0.8006 | 0.1994 | 0.0093 | 1 | 8 | 1083 |
| 35.0000 | 0.8006 | 0.1994 | 0.0093 | 0 | 2 | 1074 |
| 36.0000 | 0.8006 | 0.1994 | 0.0093 | 0 | 1072 | 1072 |

HCQ+azithromycin (AZI) Proportional Hazards Fit

Censored By: Censor

Effect Summary

Source LogWorth PValue

HCQ+AZI Buckets 51.112 0.00000


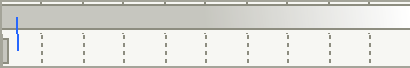
Any HCQ+AZI 0.936 0.11581

Whole Model

Number of Events 491

Number of Censorings 2021

Total Number 2512

7322.03 7356.97

AICc BIC

| **Model** | **-LogLikelihood** | **ChiSquare** | **DF** | **Prob>Chisq** |
| --- | --- | --- | --- | --- |
| Difference | 125.954 | 251.9073 | 6 | <.0001* |
| Full | 3655.000 |  |  |  |
| Reduced | 3780.954 |  |  |  |

| **Parameter Estimates** |  | | | |
| --- | --- | --- | --- | --- |
| **Term** | **Estimate** | **Std Error** | **Lower 95%** | **Upper 95%** |
| HCQ+AZI Buckets[2-1] | 1.15129187 | 0.1859765 | 0.7867846 | 1.5157991 |
| HCQ+AZI Buckets[3-2] | -1.7365693 | 0.2334183 | -2.194061 | -1.279078 |
| HCQ+AZI Buckets[4-3] | 2.01411811 | 0.2292506 | 1.5647952 | 2.463441 |
| HCQ+AZI Buckets[5-4] | 0.24356949 | 0.113005 | 0.0220838 | 0.4650552 |
| Any HCQ+AZI[NA] | 0.13193819 | 0.0698646 | -0.004994 | 0.2688704 |
| Any HCQ+AZI[No] | -0.0563469 | 0.0871531 | -0.227164 | 0.1144701 |

Confidence Intervals are Wald

Baseline Survival at mean


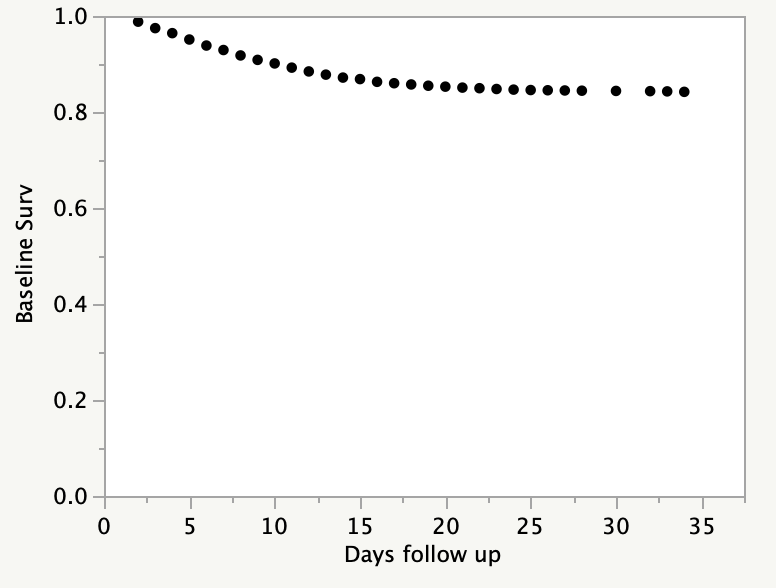


Effect Likelihood Ratio Tests

| **Source** | **Nparm** | **DF** | **L-R**  **ChiSquare** | **Prob>ChiSq** |
| --- | --- | --- | --- | --- |
| HCQ+AZI Buckets | 4 | 4 | 245.010285 | <.0001* |
| Any HCQ+AZI | 2 | 2 | 4.31164451 | 0.1158 |
| **Effect Wald Tests** |  |  |  |  |
| **Source** | **Nparm** | **DF** | **Wald ChiSquare** | **Prob>ChiSq** |
| HCQ+AZI Buckets | 4 | 4 | 171.480069 | <.0001* |
| Any HCQ+AZI | 2 | 2 | 4.42332252 | 0.1095 |

Risk Ratios

Risk Ratios for HCQ+AZI Buckets of Temp5.12.20.3.jmp

| **Level1** | **/Level2** | **Risk Ratio** | **Prob>Chisq** | **Lower 95%** | **Upper 95%** |
| --- | --- | --- | --- | --- | --- |
| 2 | 1 | 3.1622755 | <.0001* | 2.1963231 | 4.5530581 |
| 3 | 1 | 0.5569513 | 0.0282* | 0.3301938 | 0.9394326 |
| 3 | 2 | 0.1761236 | <.0001* | 0.1114632 | 0.2782938 |
| 4 | 1 | 4.1738577 | <.0001* | 2.9281076 | 5.9496066 |
| 4 | 2 | 1.3198906 | 0.0282* | 1.0301111 | 1.6911876 |
| 4 | 3 | 7.4941155 | <.0001* | 4.7816957 | 11.745157 |
| 5 | 1 | 5.3249867 | <.0001* | 3.7631271 | 7.5350853 |
| 5 | 2 | 1.6839098 | <.0001* | 1.3284664 | 2.1344553 |
| 5 | 3 | 9.5609549 | <.0001* | 6.133069 | 14.90475 |
| 5 | 4 | 1.275795 | 0.0311* | 1.0223294 | 1.5921021 |
| 1 | 2 | 0.316228 | <.0001* | 0.2196326 | 0.4553064 |
| 1 | 3 | 1.795489 | 0.0282* | 1.0644723 | 3.0285247 |
| 2 | 3 | 5.6778308 | <.0001* | 3.5933244 | 8.9715705 |
| 1 | 4 | 0.2395865 | <.0001* | 0.1680783 | 0.3415175 |
| 2 | 4 | 0.7576386 | 0.0282* | 0.5913005 | 0.970769 |
| 3 | 4 | 0.133438 | <.0001* | 0.0851415 | 0.2091308 |
| 1 | 5 | 0.1877939 | <.0001* | 0.1327125 | 0.2657364 |
| 2 | 5 | 0.593856 | <.0001* | 0.4685036 | 0.7527477 |
| 3 | 5 | 0.1045921 | <.0001* | 0.0670927 | 0.1630505 |
| 4 | 5 | 0.783825 | 0.0311* | 0.6281004 | 0.9781583 |

Risk Ratios for Any HCQ+AZI

| **Level1** | **/Level2** | **Risk Ratio** | **Prob>Chisq** | **Lower 95%** | **Upper 95%** |
| --- | --- | --- | --- | --- | --- |
| No | NA | 0.8283785 | 0.1932 | 0.6238183 | 1.1000174 |
| Yes | NA | 0.8125893 | 0.0399* | 0.6666855 | 0.9904241 |
| Yes | No | 0.9809396 | 0.8870 | 0.7522616 | 1.2791329 |
| NA | No | 1.2071776 | 0.1932 | 0.9090766 | 1.6030309 |
| NA | Yes | 1.230634 | 0.0399* | 1.0096685 | 1.4999577 |
| No | Yes | 1.0194308 | 0.8870 | 0.7817796 | 1.3293249 |

Normal approximations used for ratio confidence limits effects: HCQ+AZI Buckets of Temp5.12.20.3.jmp Any HCQ+AZI

Product-Limit Survival Fit Survival Plot


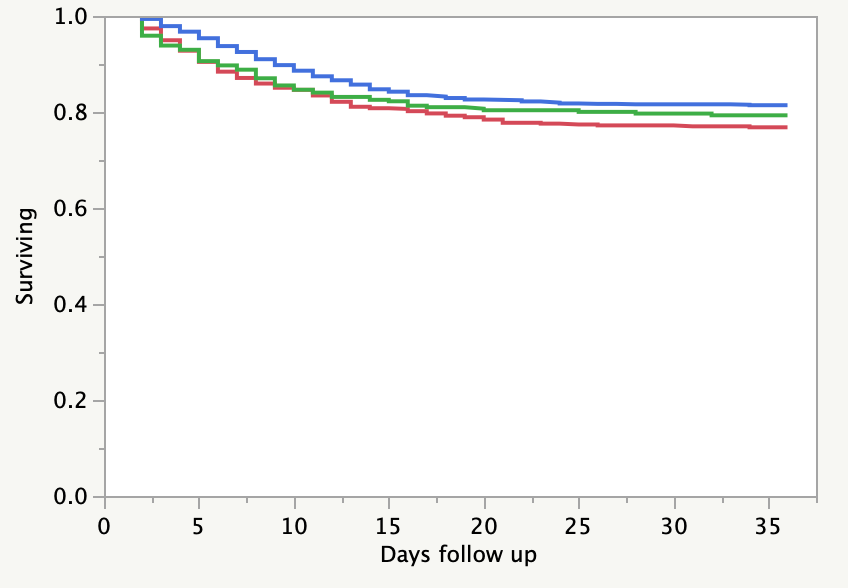

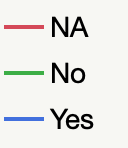


Time to event: Days follow up Censored by Censor

Censor Code 1

Grouped by Any HCQ+AZI

Summary

| **Group Number Number Mean Std Error failed censored** | | | |  |
| --- | --- | --- | --- | --- |
| NA | 156 | 541 28.1595 Biased | 0.42556 |  |
| No | 69 | 273 27.0873 Biased | 0.55289 |  |
| Yes | 266 | 1207 29.5135 Biased | 0.25566 |  |
| Combined | 491 | 2021 29.0243 Biased | 0.20721 |  |
| **Quantiles** |  |  |  |  |
| **Group** | **Median Time** | **Lower 95% Upper 95%** | **25%**  **Failures** | **75%**  **Failures** |
| NA | . | . . | . | . |
| No | . | . . | . | . |
| Yes | . | . . | . | . |
| Combined | . | . . | . | . |

Tests Between Groups

| **Test** | **ChiSquare** | **DF** | **Prob>ChiSq** |  | |
| --- | --- | --- | --- | --- | --- |
| Log-Rank | 7.1507 | 2 | 0.0280* |  |  |
| Wilcoxon | 8.3202 | 2 | 0.0156* |  |  |
| **No** |  |  |  |  |  |
| **Days follow up** | **Survival** | **Failure** | **SurvStdErr** | **Number Number failed censored** | **At Risk** |
| 0.0000 | 1.0000 | 0.0000 | 0.0000 | 0 0 | 342 |
| 1.0000 | 1.0000 | 0.0000 | 0.0000 | 0 1 | 342 |
| 2.0000 | 0.9589 | 0.0411 | 0.0107 | 14 0 | 341 |
| 3.0000 | 0.9384 | 0.0616 | 0.0130 | 7 1 | 327 |
| 4.0000 | 0.9296 | 0.0704 | 0.0139 | 3 1 | 319 |
| 5.0000 | 0.9060 | 0.0940 | 0.0158 | 8 2 | 315 |
| 6.0000 | 0.8971 | 0.1029 | 0.0165 | 3 0 | 305 |
| 7.0000 | 0.8882 | 0.1118 | 0.0171 | 3 1 | 302 |
| 8.0000 | 0.8703 | 0.1297 | 0.0182 | 6 1 | 298 |
| 9.0000 | 0.8553 | 0.1447 | 0.0191 | 5 0 | 291 |

| **Days follow up** | **Survival** | **Failure** | **SurvStdErr** | **Number failed** | **Number censored** | **At Risk** |
| --- | --- | --- | --- | --- | --- | --- |
| 10.0000 | 0.8464 | 0.1536 | 0.0196 | 3 | 0 | 286 |
| 11.0000 | 0.8404 | 0.1596 | 0.0199 | 2 | 0 | 283 |
| 12.0000 | 0.8314 | 0.1686 | 0.0204 | 3 | 1 | 281 |
| 13.0000 | 0.8314 | 0.1686 | 0.0204 | 0 | 1 | 277 |
| 14.0000 | 0.8254 | 0.1746 | 0.0207 | 2 | 3 | 276 |
| 15.0000 | 0.8223 | 0.1777 | 0.0208 | 1 | 1 | 271 |
| 16.0000 | 0.8132 | 0.1868 | 0.0212 | 3 | 4 | 269 |
| 17.0000 | 0.8101 | 0.1899 | 0.0214 | 1 | 1 | 262 |
| 18.0000 | 0.8101 | 0.1899 | 0.0214 | 0 | 3 | 260 |
| 19.0000 | 0.8069 | 0.1931 | 0.0215 | 1 | 5 | 257 |
| 20.0000 | 0.8037 | 0.1963 | 0.0217 | 1 | 5 | 251 |
| 21.0000 | 0.8037 | 0.1963 | 0.0217 | 0 | 5 | 245 |
| 22.0000 | 0.8037 | 0.1963 | 0.0217 | 0 | 3 | 240 |
| 23.0000 | 0.8037 | 0.1963 | 0.0217 | 0 | 3 | 237 |
| 25.0000 | 0.8003 | 0.1997 | 0.0219 | 1 | 0 | 234 |
| 26.0000 | 0.8003 | 0.1997 | 0.0219 | 0 | 5 | 233 |
| 27.0000 | 0.8003 | 0.1997 | 0.0219 | 0 | 1 | 228 |
| 28.0000 | 0.7967 | 0.2033 | 0.0221 | 1 | 4 | 227 |
| 29.0000 | 0.7967 | 0.2033 | 0.0221 | 0 | 1 | 222 |
| 31.0000 | 0.7967 | 0.2033 | 0.0221 | 0 | 1 | 221 |
| 32.0000 | 0.7931 | 0.2069 | 0.0222 | 1 | 3 | 220 |
| 33.0000 | 0.7931 | 0.2069 | 0.0222 | 0 | 1 | 216 |
| 34.0000 | 0.7931 | 0.2069 | 0.0222 | 0 | 2 | 215 |
| 35.0000 | 0.7931 | 0.2069 | 0.0222 | 0 | 2 | 213 |
| 36.0000 | 0.7931 | 0.2069 | 0.0222 | 0 | 211 | 211 |

Yes

| **Days follow up** | **Survival** | **Failure** | **SurvStdErr** | **Number failed** | **Number censored** | **At Risk** |
| --- | --- | --- | --- | --- | --- | --- |
| 0.0000 | 1.0000 | 0.0000 | 0.0000 | 0 | 0 | 1473 |
| 1.0000 | 1.0000 | 0.0000 | 0.0000 | 0 | 1 | 1473 |
| 2.0000 | 0.9939 | 0.0061 | 0.0020 | 9 | 2 | 1472 |
| 3.0000 | 0.9789 | 0.0211 | 0.0037 | 22 | 3 | 1461 |
| 4.0000 | 0.9673 | 0.0327 | 0.0046 | 17 | 0 | 1436 |
| 5.0000 | 0.9537 | 0.0463 | 0.0055 | 20 | 0 | 1419 |
| 6.0000 | 0.9373 | 0.0627 | 0.0063 | 24 | 2 | 1399 |
| 7.0000 | 0.9250 | 0.0750 | 0.0069 | 18 | 3 | 1373 |

| **Days follow up** | **Survival** | **Failure** | **SurvStdErr** | **Number failed** | **Number censored** | **At Risk** |
| --- | --- | --- | --- | --- | --- | --- |
| 8.0000 | 0.9100 | 0.0900 | 0.0075 | 22 | 0 | 1352 |
| 9.0000 | 0.8977 | 0.1023 | 0.0079 | 18 | 2 | 1330 |
| 10.0000 | 0.8860 | 0.1140 | 0.0083 | 17 | 2 | 1310 |
| 11.0000 | 0.8744 | 0.1256 | 0.0087 | 17 | 6 | 1291 |
| 12.0000 | 0.8661 | 0.1339 | 0.0089 | 12 | 5 | 1268 |
| 13.0000 | 0.8571 | 0.1429 | 0.0092 | 13 | 11 | 1251 |
| 14.0000 | 0.8473 | 0.1527 | 0.0094 | 14 | 24 | 1227 |
| 15.0000 | 0.8423 | 0.1577 | 0.0095 | 7 | 24 | 1189 |
| 16.0000 | 0.8350 | 0.1650 | 0.0097 | 10 | 13 | 1158 |
| 17.0000 | 0.8321 | 0.1679 | 0.0098 | 4 | 15 | 1135 |
| 18.0000 | 0.8291 | 0.1709 | 0.0099 | 4 | 28 | 1116 |
| 19.0000 | 0.8261 | 0.1739 | 0.0100 | 4 | 27 | 1084 |
| 20.0000 | 0.8253 | 0.1747 | 0.0100 | 1 | 18 | 1053 |
| 21.0000 | 0.8245 | 0.1755 | 0.0100 | 1 | 27 | 1034 |
| 22.0000 | 0.8220 | 0.1780 | 0.0101 | 3 | 27 | 1006 |
| 23.0000 | 0.8195 | 0.1805 | 0.0102 | 3 | 16 | 976 |
| 24.0000 | 0.8178 | 0.1822 | 0.0102 | 2 | 12 | 957 |
| 25.0000 | 0.8169 | 0.1831 | 0.0102 | 1 | 5 | 943 |
| 26.0000 | 0.8169 | 0.1831 | 0.0102 | 0 | 17 | 937 |
| 27.0000 | 0.8160 | 0.1840 | 0.0103 | 1 | 7 | 920 |
| 28.0000 | 0.8160 | 0.1840 | 0.0103 | 0 | 13 | 912 |
| 29.0000 | 0.8160 | 0.1840 | 0.0103 | 0 | 6 | 899 |
| 30.0000 | 0.8160 | 0.1840 | 0.0103 | 0 | 6 | 893 |
| 31.0000 | 0.8160 | 0.1840 | 0.0103 | 0 | 3 | 887 |
| 32.0000 | 0.8160 | 0.1840 | 0.0103 | 0 | 7 | 884 |
| 33.0000 | 0.8151 | 0.1849 | 0.0103 | 1 | 6 | 877 |
| 34.0000 | 0.8142 | 0.1858 | 0.0103 | 1 | 7 | 870 |
| 35.0000 | 0.8142 | 0.1858 | 0.0103 | 0 | 1 | 862 |
| 36.0000 | 0.8142 | 0.1858 | 0.0103 | 0 | 861 | 861 |

HCQ & AZI Factorial Proportional Hazards Fit

Censored By: Censor

Effect Summary

Source LogWorth PValue

Factorial Buckets 31.211 0.00000


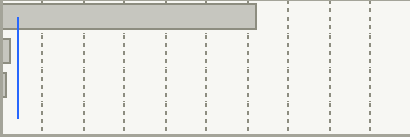
Any AZI*Any HCQ 1.027 0.09389

Any AZI 0.556 0.27781 ^

Any HCQ 0.082 0.82884 ^

Whole Model

Number of Events 491

Number of Censorings 2021

Total Number 2512

7415.88 7456.63

AICc BIC

| **Model** | **-LogLikelihood** | | **ChiSquare** | | **DF** | **Prob>Chisq** |  |
| --- | --- | --- | --- | --- | --- | --- | --- |
| Difference | 80.038 | | 160.0757 | | 7 | <.0001* |  |
| Full | 3700.916 | |  | |  |  |  |
| Reduced | 3780.954 | |  | |  |  |  |
| **Parameter Estimates** | |  | |  | |  |  |
| **Term** | | **Estimate** | | **Std Error** | | **Lower 95%** | **Upper 95%** |
| Factorial Buckets[2-1] | | 0.19093277 | | 0.1431142 | | -0.089566 | 0.4714315 |
| Factorial Buckets[3-2] | | -0.3789811 | | 0.1506424 | | -0.674235 | -0.083727 |
| Factorial Buckets[4-3] | | -0.8475834 | | 0.2069058 | | -1.253111 | -0.442056 |
| Factorial Buckets[5-4] | | 1.86201624 | | 0.1867533 | | 1.4959864 | 2.228046 |
| Any HCQ[No] | | -0.0117258 | | 0.0543284 | | -0.118208 | 0.094756 |
| Any AZI[No] | | 0.05889537 | | 0.0543215 | | -0.047573 | 0.1653636 |
| Any AZI[No]*Any HCQ[No] | | -0.0916134 | | 0.0542753 | | -0.197991 | 0.0147643 |

Confidence Intervals are Wald

Baseline Survival at mean


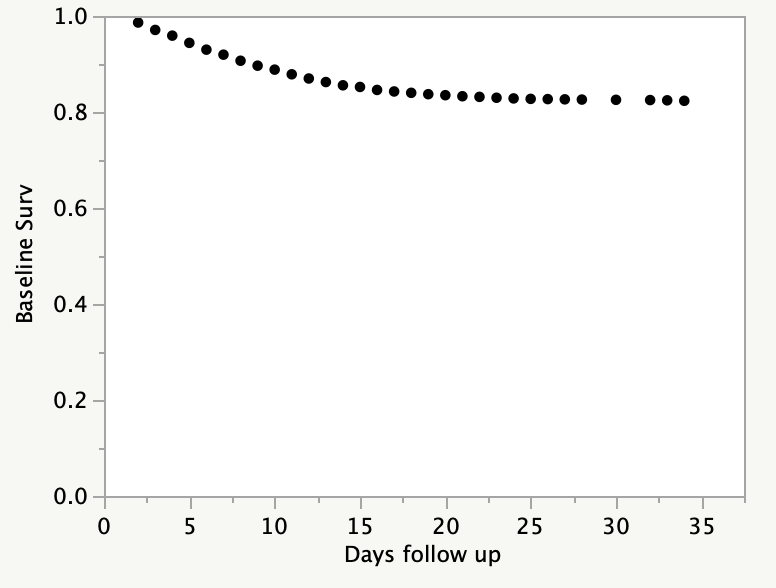


Effect Likelihood Ratio Tests

| **Source** | **Nparm** | **DF** | **L-R**  **ChiSquare** | **Prob>ChiSq** |
| --- | --- | --- | --- | --- |
| Factorial Buckets | 4 | 4 | 152.423648 | <.0001* |
| Any HCQ | 1 | 1 | 0.04673709 | 0.8288 |
| Any AZI | 1 | 1 | 1.17774886 | 0.2778 |
| Any AZI*Any HCQ | 1 | 1 | 2.80638268 | 0.0939 |
| **Effect Wald Tests** |  |  |  |  |
| **Source** | **Nparm** | **DF** | **Wald ChiSquare** | **Prob>ChiSq** |
| Factorial Buckets | 4 | 4 | 137.69672 | <.0001* |
| Any HCQ | 1 | 1 | 0.04658323 | 0.8291 |
| Any AZI | 1 | 1 | 1.17548953 | 0.2783 |
| Any AZI*Any HCQ | 1 | 1 | 2.84913508 | 0.0914 |

Risk Ratios

Risk Ratios for Factorial Buckets

| **Level1** | **/Level2** | **Risk Ratio** | **Prob>Chisq** | **Lower 95%** | **Upper 95%** |
| --- | --- | --- | --- | --- | --- |
| 2 | 1 | 1.2103781 | 0.1822 | 0.9143279 | 1.6022863 |
| 3 | 1 | 0.8285746 | 0.2296 | 0.6096577 | 1.1261007 |
| 3 | 2 | 0.6845585 | 0.0119* | 0.5095462 | 0.9196819 |
| 4 | 1 | 0.355002 | <.0001* | 0.2391409 | 0.5269966 |
| 4 | 2 | 0.2932985 | <.0001* | 0.1993518 | 0.4315184 |
| 4 | 3 | 0.4284491 | <.0001* | 0.2856148 | 0.6427139 |
| 5 | 1 | 2.2850421 | <.0001* | 1.7755556 | 2.9407231 |
| 5 | 2 | 1.8878747 | <.0001* | 1.4876869 | 2.3957129 |
| 5 | 3 | 2.7577988 | <.0001* | 2.107964 | 3.607962 |
| 5 | 4 | 6.4367016 | <.0001* | 4.4637376 | 9.2817123 |
| 1 | 2 | 0.8261881 | 0.1822 | 0.6241082 | 1.0936995 |
| 1 | 3 | 1.2068919 | 0.2296 | 0.8880201 | 1.6402647 |
| 2 | 3 | 1.4607955 | 0.0119* | 1.0873325 | 1.9625307 |
| 1 | 4 | 2.8168854 | <.0001* | 1.8975456 | 4.181635 |
| 2 | 4 | 3.4094963 | <.0001* | 2.3173983 | 5.0162569 |
| 3 | 4 | 2.3339998 | <.0001* | 1.5559022 | 3.5012192 |
| 1 | 5 | 0.4376287 | <.0001* | 0.3400524 | 0.563204 |
| 2 | 5 | 0.5296962 | <.0001* | 0.4174123 | 0.6721844 |
| 3 | 5 | 0.362608 | <.0001* | 0.2771648 | 0.4743914 |
| 4 | 5 | 0.1553591 | <.0001* | 0.1077387 | 0.2240275 |

Risk Ratios for Any HCQ

| **Level1** | **/Level2** | **Risk Ratio** | **Prob>Chisq** | **Lower 95%** | **Upper 95%** |
| --- | --- | --- | --- | --- | --- |
| Yes | No | 1.0237287 | 0.8291 | 0.8273628 | 1.2667 |
| No | Yes | 0.9768213 | 0.8291 | 0.7894529 | 1.2086596 |

Risk Ratios for Any AZI

| **Level1** | **/Level2** | **Risk Ratio** | **Prob>Chisq** | **Lower 95%** | **Upper 95%** |
| --- | --- | --- | --- | --- | --- |
| Yes | No | 0.888882 | 0.2783 | 0.7184012 | 1.099819 |
| No | Yes | 1.1250087 | 0.2783 | 0.9092405 | 1.3919799 |

Normal approximations used for ratio confidence limits effects: Factorial Buckets Any HCQ Any AZI

Product-Limit Survival Fit Survival Plot


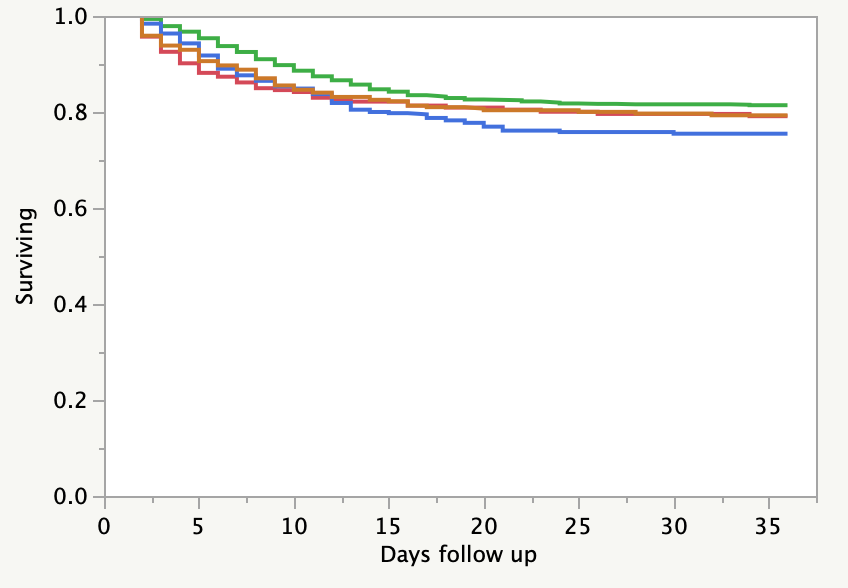

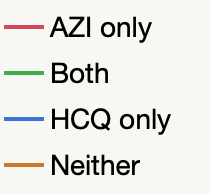


Time to event: Days follow up Censored by Censor

Censor Code 1 Grouped by Group

Summary

| **Group Number Number Mean Std Error failed censored** | | | |  |
| --- | --- | --- | --- | --- |
| AZI only | 52 | 204 28.4911 Biased | 0.71306 |  |
| Both | 266 | 1207 29.5135 Biased | 0.25566 |  |
| HCQ only | 104 | 337 24.9458 Biased | 0.45275 |  |
| Neither | 69 | 273 27.0873 Biased | 0.55289 |  |
| Combined | 491 | 2021 29.0243 Biased | 0.20721 |  |
| **Quantiles** |  |  |  |  |
| **Group** | **Median Time** | **Lower 95% Upper 95%** | **25%**  **Failures** | **75%**  **Failures** |
| AZI only | . | . . | . | . |
| Both | . | . . | . | . |
| HCQ only | . | . . | . | . |
| Neither | . | . . | . | . |
| Combined | . | . . | . | . |

Tests Between Groups

| **Test** | **ChiSquare** | **DF** | **Prob>ChiSq** |  | |
| --- | --- | --- | --- | --- | --- |
| Log-Rank | 8.0426 | 3 | 0.0451* |  |  |
| Wilcoxon | 8.8227 | 3 | 0.0317* |  |  |
| **AZI only** |  |  |  |  |  |
| **Days follow up** | **Survival** | **Failure** | **SurvStdErr** | **Number Number failed censored** | **At Risk** |
| 0.0000 | 1.0000 | 0.0000 | 0.0000 | 0 0 | 256 |
| 2.0000 | 0.9570 | 0.0430 | 0.0127 | 11 3 | 256 |
| 3.0000 | 0.9254 | 0.0746 | 0.0165 | 8 2 | 242 |
| 4.0000 | 0.9015 | 0.0985 | 0.0187 | 6 0 | 232 |
| 5.0000 | 0.8815 | 0.1185 | 0.0203 | 5 0 | 226 |
| 6.0000 | 0.8735 | 0.1265 | 0.0209 | 2 0 | 221 |
| 7.0000 | 0.8616 | 0.1384 | 0.0217 | 3 0 | 219 |
| 8.0000 | 0.8496 | 0.1504 | 0.0225 | 3 0 | 216 |
| 9.0000 | 0.8456 | 0.1544 | 0.0227 | 1 0 | 213 |

| **Days follow up** | **Survival** | **Failure** | **SurvStdErr** | **Number failed** | **Number censored** | **At Risk** |
| --- | --- | --- | --- | --- | --- | --- |
| 10.0000 | 0.8416 | 0.1584 | 0.0230 | 1 | 0 | 212 |
| 11.0000 | 0.8297 | 0.1703 | 0.0237 | 3 | 1 | 211 |
| 12.0000 | 0.8257 | 0.1743 | 0.0239 | 1 | 1 | 207 |
| 13.0000 | 0.8216 | 0.1784 | 0.0241 | 1 | 3 | 205 |
| 15.0000 | 0.8216 | 0.1784 | 0.0241 | 0 | 2 | 201 |
| 16.0000 | 0.8134 | 0.1866 | 0.0246 | 2 | 2 | 199 |
| 18.0000 | 0.8092 | 0.1908 | 0.0248 | 1 | 3 | 195 |
| 19.0000 | 0.8092 | 0.1908 | 0.0248 | 0 | 2 | 191 |
| 21.0000 | 0.8049 | 0.1951 | 0.0250 | 1 | 2 | 189 |
| 22.0000 | 0.8049 | 0.1951 | 0.0250 | 0 | 3 | 186 |
| 23.0000 | 0.8005 | 0.1995 | 0.0253 | 1 | 1 | 183 |
| 24.0000 | 0.8005 | 0.1995 | 0.0253 | 0 | 2 | 181 |
| 25.0000 | 0.8005 | 0.1995 | 0.0253 | 0 | 3 | 179 |
| 26.0000 | 0.7960 | 0.2040 | 0.0255 | 1 | 2 | 176 |
| 30.0000 | 0.7960 | 0.2040 | 0.0255 | 0 | 2 | 173 |
| 31.0000 | 0.7960 | 0.2040 | 0.0255 | 0 | 1 | 171 |
| 34.0000 | 0.7913 | 0.2087 | 0.0258 | 1 | 0 | 170 |
| 36.0000 | 0.7913 | 0.2087 | 0.0258 | 0 | 169 | 169 |

Both

| **Days follow up** | **Survival** | **Failure** | **SurvStdErr** | **Number failed** | **Number censored** | **At Risk** |
| --- | --- | --- | --- | --- | --- | --- |
| 0.0000 | 1.0000 | 0.0000 | 0.0000 | 0 | 0 | 1473 |
| 1.0000 | 1.0000 | 0.0000 | 0.0000 | 0 | 1 | 1473 |
| 2.0000 | 0.9939 | 0.0061 | 0.0020 | 9 | 2 | 1472 |
| 3.0000 | 0.9789 | 0.0211 | 0.0037 | 22 | 3 | 1461 |
| 4.0000 | 0.9673 | 0.0327 | 0.0046 | 17 | 0 | 1436 |
| 5.0000 | 0.9537 | 0.0463 | 0.0055 | 20 | 0 | 1419 |
| 6.0000 | 0.9373 | 0.0627 | 0.0063 | 24 | 2 | 1399 |
| 7.0000 | 0.9250 | 0.0750 | 0.0069 | 18 | 3 | 1373 |
| 8.0000 | 0.9100 | 0.0900 | 0.0075 | 22 | 0 | 1352 |
| 9.0000 | 0.8977 | 0.1023 | 0.0079 | 18 | 2 | 1330 |
| 10.0000 | 0.8860 | 0.1140 | 0.0083 | 17 | 2 | 1310 |
| 11.0000 | 0.8744 | 0.1256 | 0.0087 | 17 | 6 | 1291 |
| 12.0000 | 0.8661 | 0.1339 | 0.0089 | 12 | 5 | 1268 |
| 13.0000 | 0.8571 | 0.1429 | 0.0092 | 13 | 11 | 1251 |
| 14.0000 | 0.8473 | 0.1527 | 0.0094 | 14 | 24 | 1227 |

| **Days follow up** | **Survival** | **Failure** | **SurvStdErr** | **Number failed** | **Number censored** | **At Risk** |
| --- | --- | --- | --- | --- | --- | --- |
| 15.0000 | 0.8423 | 0.1577 | 0.0095 | 7 | 24 | 1189 |
| 16.0000 | 0.8350 | 0.1650 | 0.0097 | 10 | 13 | 1158 |
| 17.0000 | 0.8321 | 0.1679 | 0.0098 | 4 | 15 | 1135 |
| 18.0000 | 0.8291 | 0.1709 | 0.0099 | 4 | 28 | 1116 |
| 19.0000 | 0.8261 | 0.1739 | 0.0100 | 4 | 27 | 1084 |
| 20.0000 | 0.8253 | 0.1747 | 0.0100 | 1 | 18 | 1053 |
| 21.0000 | 0.8245 | 0.1755 | 0.0100 | 1 | 27 | 1034 |
| 22.0000 | 0.8220 | 0.1780 | 0.0101 | 3 | 27 | 1006 |
| 23.0000 | 0.8195 | 0.1805 | 0.0102 | 3 | 16 | 976 |
| 24.0000 | 0.8178 | 0.1822 | 0.0102 | 2 | 12 | 957 |
| 25.0000 | 0.8169 | 0.1831 | 0.0102 | 1 | 5 | 943 |
| 26.0000 | 0.8169 | 0.1831 | 0.0102 | 0 | 17 | 937 |
| 27.0000 | 0.8160 | 0.1840 | 0.0103 | 1 | 7 | 920 |
| 28.0000 | 0.8160 | 0.1840 | 0.0103 | 0 | 13 | 912 |
| 29.0000 | 0.8160 | 0.1840 | 0.0103 | 0 | 6 | 899 |
| 30.0000 | 0.8160 | 0.1840 | 0.0103 | 0 | 6 | 893 |
| 31.0000 | 0.8160 | 0.1840 | 0.0103 | 0 | 3 | 887 |
| 32.0000 | 0.8160 | 0.1840 | 0.0103 | 0 | 7 | 884 |
| 33.0000 | 0.8151 | 0.1849 | 0.0103 | 1 | 6 | 877 |
| 34.0000 | 0.8142 | 0.1858 | 0.0103 | 1 | 7 | 870 |
| 35.0000 | 0.8142 | 0.1858 | 0.0103 | 0 | 1 | 862 |
| 36.0000 | 0.8142 | 0.1858 | 0.0103 | 0 | 861 | 861 |

HCQ only

| **Days follow up** | **Survival** | **Failure** | **SurvStdErr** | **Number failed** | **Number censored** | **At Risk** |
| --- | --- | --- | --- | --- | --- | --- |
| 0.0000 | 1.0000 | 0.0000 | 0.0000 | 0 | 0 | 441 |
| 0.0000 | 1.0000 | 0.0000 | 0.0000 | 0 | 1 | 441 |
| 1.0000 | 1.0000 | 0.0000 | 0.0000 | 0 | 1 | 440 |
| 2.0000 | 0.9841 | 0.0159 | 0.0060 | 7 | 1 | 439 |
| 3.0000 | 0.9635 | 0.0365 | 0.0090 | 9 | 0 | 431 |
| 4.0000 | 0.9430 | 0.0570 | 0.0111 | 9 | 2 | 422 |
| 5.0000 | 0.9177 | 0.0823 | 0.0131 | 11 | 0 | 411 |
| 6.0000 | 0.8902 | 0.1098 | 0.0150 | 12 | 0 | 400 |
| 7.0000 | 0.8764 | 0.1236 | 0.0157 | 6 | 0 | 388 |
| 8.0000 | 0.8650 | 0.1350 | 0.0164 | 5 | 1 | 382 |
| 9.0000 | 0.8534 | 0.1466 | 0.0169 | 5 | 1 | 376 |

| **Days follow up** | **Survival** | **Failure** | **SurvStdErr** | **Number failed** | **Number censored** | **At Risk** |
| --- | --- | --- | --- | --- | --- | --- |
| 10.0000 | 0.8488 | 0.1512 | 0.0171 | 2 | 0 | 370 |
| 11.0000 | 0.8373 | 0.1627 | 0.0177 | 5 | 1 | 368 |
| 12.0000 | 0.8188 | 0.1812 | 0.0185 | 8 | 3 | 362 |
| 13.0000 | 0.8048 | 0.1952 | 0.0190 | 6 | 8 | 351 |
| 14.0000 | 0.8000 | 0.2000 | 0.0192 | 2 | 8 | 337 |
| 15.0000 | 0.7976 | 0.2024 | 0.0193 | 1 | 4 | 327 |
| 16.0000 | 0.7951 | 0.2049 | 0.0194 | 1 | 3 | 322 |
| 17.0000 | 0.7876 | 0.2124 | 0.0197 | 3 | 3 | 318 |
| 18.0000 | 0.7826 | 0.2174 | 0.0199 | 2 | 5 | 312 |
| 19.0000 | 0.7774 | 0.2226 | 0.0201 | 2 | 9 | 305 |
| 20.0000 | 0.7695 | 0.2305 | 0.0204 | 3 | 11 | 294 |
| 21.0000 | 0.7612 | 0.2388 | 0.0207 | 3 | 8 | 280 |
| 22.0000 | 0.7612 | 0.2388 | 0.0207 | 0 | 7 | 269 |
| 23.0000 | 0.7612 | 0.2388 | 0.0207 | 0 | 10 | 262 |
| 24.0000 | 0.7582 | 0.2418 | 0.0208 | 1 | 8 | 252 |
| 25.0000 | 0.7582 | 0.2418 | 0.0208 | 0 | 10 | 243 |
| 26.0000 | 0.7582 | 0.2418 | 0.0208 | 0 | 6 | 233 |
| 27.0000 | 0.7582 | 0.2418 | 0.0208 | 0 | 3 | 227 |
| 28.0000 | 0.7582 | 0.2418 | 0.0208 | 0 | 2 | 224 |
| 29.0000 | 0.7582 | 0.2418 | 0.0208 | 0 | 1 | 222 |
| 30.0000 | 0.7548 | 0.2452 | 0.0210 | 1 | 3 | 221 |
| 32.0000 | 0.7548 | 0.2452 | 0.0210 | 0 | 2 | 217 |
| 33.0000 | 0.7548 | 0.2452 | 0.0210 | 0 | 2 | 215 |
| 34.0000 | 0.7548 | 0.2452 | 0.0210 | 0 | 1 | 213 |
| 35.0000 | 0.7548 | 0.2452 | 0.0210 | 0 | 1 | 212 |
| 36.0000 | 0.7548 | 0.2452 | 0.0210 | 0 | 211 | 211 |

Neither

| **Days follow up** | **Survival** | **Failure** | **SurvStdErr** | **Number failed** | **Number censored** | **At Risk** |
| --- | --- | --- | --- | --- | --- | --- |
| 0.0000 | 1.0000 | 0.0000 | 0.0000 | 0 | 0 | 342 |
| 1.0000 | 1.0000 | 0.0000 | 0.0000 | 0 | 1 | 342 |
| 2.0000 | 0.9589 | 0.0411 | 0.0107 | 14 | 0 | 341 |
| 3.0000 | 0.9384 | 0.0616 | 0.0130 | 7 | 1 | 327 |
| 4.0000 | 0.9296 | 0.0704 | 0.0139 | 3 | 1 | 319 |
| 5.0000 | 0.9060 | 0.0940 | 0.0158 | 8 | 2 | 315 |
| 6.0000 | 0.8971 | 0.1029 | 0.0165 | 3 | 0 | 305 |

| **Days follow up** | **Survival** | **Failure** | **SurvStdErr** | **Number failed** | **Number censored** | **At Risk** |
| --- | --- | --- | --- | --- | --- | --- |
| 7.0000 | 0.8882 | 0.1118 | 0.0171 | 3 | 1 | 302 |
| 8.0000 | 0.8703 | 0.1297 | 0.0182 | 6 | 1 | 298 |
| 9.0000 | 0.8553 | 0.1447 | 0.0191 | 5 | 0 | 291 |
| 10.0000 | 0.8464 | 0.1536 | 0.0196 | 3 | 0 | 286 |
| 11.0000 | 0.8404 | 0.1596 | 0.0199 | 2 | 0 | 283 |
| 12.0000 | 0.8314 | 0.1686 | 0.0204 | 3 | 1 | 281 |
| 13.0000 | 0.8314 | 0.1686 | 0.0204 | 0 | 1 | 277 |
| 14.0000 | 0.8254 | 0.1746 | 0.0207 | 2 | 3 | 276 |
| 15.0000 | 0.8223 | 0.1777 | 0.0208 | 1 | 1 | 271 |
| 16.0000 | 0.8132 | 0.1868 | 0.0212 | 3 | 4 | 269 |
| 17.0000 | 0.8101 | 0.1899 | 0.0214 | 1 | 1 | 262 |
| 18.0000 | 0.8101 | 0.1899 | 0.0214 | 0 | 3 | 260 |
| 19.0000 | 0.8069 | 0.1931 | 0.0215 | 1 | 5 | 257 |
| 20.0000 | 0.8037 | 0.1963 | 0.0217 | 1 | 5 | 251 |
| 21.0000 | 0.8037 | 0.1963 | 0.0217 | 0 | 5 | 245 |
| 22.0000 | 0.8037 | 0.1963 | 0.0217 | 0 | 3 | 240 |
| 23.0000 | 0.8037 | 0.1963 | 0.0217 | 0 | 3 | 237 |
| 25.0000 | 0.8003 | 0.1997 | 0.0219 | 1 | 0 | 234 |
| 26.0000 | 0.8003 | 0.1997 | 0.0219 | 0 | 5 | 233 |
| 27.0000 | 0.8003 | 0.1997 | 0.0219 | 0 | 1 | 228 |
| 28.0000 | 0.7967 | 0.2033 | 0.0221 | 1 | 4 | 227 |
| 29.0000 | 0.7967 | 0.2033 | 0.0221 | 0 | 1 | 222 |
| 31.0000 | 0.7967 | 0.2033 | 0.0221 | 0 | 1 | 221 |
| 32.0000 | 0.7931 | 0.2069 | 0.0222 | 1 | 3 | 220 |
| 33.0000 | 0.7931 | 0.2069 | 0.0222 | 0 | 1 | 216 |
| 34.0000 | 0.7931 | 0.2069 | 0.0222 | 0 | 2 | 215 |
| 35.0000 | 0.7931 | 0.2069 | 0.0222 | 0 | 2 | 213 |
| 36.0000 | 0.7931 | 0.2069 | 0.0222 | 0 | 211 | 211 |
